# Effect of rosmarinic acid on differentiation and mineralization of MC3T3-E1 osteoblastic cells on titanium surface

**DOI:** 10.1080/19768354.2021.1886987

**Published:** 2021-02-18

**Authors:** Moon-Jin Jeong, Do-Seon Lim, Sung Ok Kim, Cheol Park, Yung Hyun Choi, Soon-Jeong Jeong

**Affiliations:** a Department of Oral Histology and Developmental Biology, School of Dentistry, Chosun University, Gwangju, Korea; b Department of Dental Hygiene, Graduate School of Public Health Science, Eulji University, Seongnam, Korea; c Department of Food Science and Biotechnology, College of Engineering, Kyungsung University, Busan, Korea; d College of Liberal Studies, Division of Basic Sciences, Dong-eui University, Busan, Korea; e Department of Biochemistry, College of Korean Medicine, Dong-eui University, Busan, Korea; f Department of Dental Hygiene, College of Health Science, Youngsan University, Yangsan, Korea; g Institute of Basic Science for Well-Aging, Youngsan University, Yangsan, Korea

**Keywords:** Differentiation, MC3T3-E1 osteoblasts, mineralization, osseointegration, rosmarinic acid, titanium

## Abstract

Titanium (Ti) is a widely used biomaterial for dental implants because of its outstanding biocompatibility for hard tissues. Osseointegration, the interaction between implanted biomaterials and living cells in bone, is essential for successful implantation. Rosmarinic acid (RA) is a plant-derived phytochemical with low toxicity and side effects and has various effects that can be applied as a therapeutic substance. The MC3T3-E1 osteoblastic cells on the Ti surface in medium with or without 14 μg/ml RA were used to test RA effects on osteoblast differentiation, cell viability and mineralization during differentiation. RA treatment increased osteoblast differentiation, cell viability and mineralization in MC3T3-E1 osteoblastic cells on Ti surface during differentiation, upregulating Runx-2 and OPG, but downregulating RANKL. This study suggest that RA should be applied as an effective functional and therapeutic substance to enhance osseointegration of osteoblast cells by increasing differentiation, mineralization, and bone formation through the RANKL/RANK/OPG pathway during the differentiation in MC3T3-E1 osteoblastic cells on the Ti surface.

## Introduction

Bones are dynamic tissues that regenerate throughout life through a balance between the activity of osteoblasts and osteoclasts (Lee et al. 2015; Fernandes et al. 2018). Osteoblasts produce and mineralize osteoid, an organic mixture for new bone formation and osteoclasts are responsible for bone absorption (Ryoo et al. 2006; Lee et al. 2015). Osteoblasts participate in bone formation by producing bone matrix, as well as play an important role in osteoclast differentiation (Hofbauer et al. 2000; Choi et al. 2007). All processes of bone formation depend heavily on the interaction between osteogenic cells and surrounding tissues including the extracellular matrix (Dejaeger et al. 2017). Bone can be regenerated to repair small damage, but in case of large damage above the critical size, biomaterials should be added to the damaged area to help repair (Fernandes et al. 2018). Titanium (Ti) and Ti alloys are widely used biomaterials for implants because of their biocompatibility for hardened tissues such as bone and teeth (Jeong et al. 2015; Jeong and Jeong 2016; Rossi et al. 2017). For many years, prosthetic dentistry and orthopedic surgeons have used Ti and Ti alloys as biomaterials for restoration of damaged bones and loss of function (Jeong et al. 2015; Jeong and Jeong 2016; Rossi et al. 2017). As a first step in the successful implantation of biomaterials, biomaterials should recruit preosteoblasts into the damaged area and induce proliferation of those recruited cells. The next step is the differentiation of proliferated preosteoblasts into osteoblasts through a modulated signaling pathway and, then those differentiated cells undergo mineralization to induce osseointegration and bone formation (Fernandes et al. 2018). Increasing mineral deposition during mineralization is an important factor in indicating viability of cells and increasing osseointegration between biomaterial and bone (Jeong and Jeong 2016). Osseointegration means direct functional and structural interaction between implanted biomaterials and living bones (Jeong and Jeong 2016). Osseointegration between implanted biomaterial and bone is essential for successful implantation and is an important determinant of implant success (Choi et al. 2016). Although the success rate of dental implants using Ti and Ti alloys is high due to their excellent mechanical properties and biocompatibility, Ti-base dental implant failures still occur due to the release of metal ions, periodontal disease, osteoporosis, diabetes, local bone loss around the patient’s teeth and delayed wound healing (Choi et al. 2016; Ma et al. 2017). Various attempts and studies have been made to resolve the problem of dental implant failure due to incomplete osseointegration between bone and Ti and Ti alloys biomaterials and to improve osseointegration through differentiation and mineralization into osteoblasts (Jeong and Jeong 2016). Although there has been some progress in the interactions between osteoblasts and biomaterials due to various attempts to improve osseointegration, much remains unknown (Zambuzzi et al. 2011; Gemini-Piperni et al. 2014).

Rosmarinic acid (RA) is a plant-derived phytochemical with low toxicity and side effects and has anti-oxidative, anti-bacterial, anti-viral, anti-inflammatory and anti-mutagen activity, which is highly applicable as a therapeutic substance for acute and chronic diseases (Kim et al. 2008; Moon et al. 2010; Lee and Han 2012; Kim et al. 2018). Although RA has been reported to prevent osteoporosis (Lee et al. 2015; Kang et al. 2017) and metastasis from breast cancer cells to bone tissue (Xu and Jiang 2010), there is no study of RA effects on osseointegration determined by the differentiation and mineralization of MC3T3-E1 osteoblastic cells on Ti surface for successful Ti-based dental implants. The purpose of this study was to identify RA effects on differentiation into osteoblasts and mineralization of MC3T3-E1 osteoblastic cells on the Ti surface and the related signaling pathways, and to confirm the applicability of RA as a functional and therapeutic substance to improve osseointegration between Ti and Ti alloy materials and osteoblasts.

## Materials and methods

### Titanium samples

15 mm, 20 mm, and 48 mm diameter polished pure Ti discs with a thickness of 2 mm were prepared according to the previously published method (Jeong et al. 2015) and used for the experiment.

### Cell culture and differentiation with RA

MC3T3-E1 cells, an osteoblastic cell line derived from mouse calvaria, were cultured in alpha-modified eagle’s medium (α-MEM; WelGENE Inc., Daegu, Republic of Korea) containing 10% fetal bovine serum (FBS, WelGENE Inc.) and 1% antibiotic antimycotic solution (WelGENE Inc.). The cells were transferred to a Ti surface and changed to a differentiation medium (α-MEM supplemented with 5% FBS, 10 mM β-glycerol phosphate and 50 μg/ml ascorbic acid) with or without 14 μg/ml RA (Sigma-Aldrich Chemical Co., St. Louis, MO, USA) after 24 h. The cells were placed into a humidified chamber and maintained in an atmosphere containing 5% CO_2_ at 37°C.

### Cell viability assay

The MC3T3-E1 cell plated on the Ti discs (3 × 105 cells/ml) were incubated for 4, 7 and 10 days in differentiation medium with or without 14 μg/ml RA. An 3-(4,5-dimethylthiazol-2-yl)-2,5-diphenyltetrazolium bromide (MTT; Sigma-Aldrich Chemical Co.) assay was performed to examine the cell viability as described previously (Jeong et al. 2015).

### Extraction of total RNA and reverse-transcription polymerase chain reaction (RT–PCR)

The MC3T3-E1 cells plated on the Ti discs (1 × 10^6^ cells/ml) were incubated for 4, 7 and 10 days in differentiation medium with or without RA. The total RNA was extracted from cells using RiboEXTM reagent (GeneAll Co., Seoul, Republic of Korea) according to the manufacturer’s instructions. A 1㎍ sample of total RNA was used to synthesize the complementary DNA (cDNA) with RT Premix (GeNet Bio Co., Daejeon, Republic of Korea). The PCR reaction was conducted in a thermocycler (Takara Bio Inc., Shiga, Japan) after adding 1 μl of cDNA and the gene specific primers (Table 1) to the PCR premix (GeneAll Co.). The annealing temperature for each primer and number of cycles were as follows: Alkaline phosphatase (ALP), 62˚C and 35 cycles; Bone sialoprotein (BSP), 60˚C and 35 cycles; Dentin sialophosphoprotein (DSPP), 55˚C and 35 cycles; Dentin matrix acidic phosphoprotein 1 (DMP-1), 55˚C and 35 cycles; Osteocalcin (OCN), 66˚C and 27 cycles; Osteonectin (ON), 63˚C and 35 cycles; Collagen type 1 (Col I), 50˚C and 35 cycles; Runt-related transcription factor 2 (Runx-2), 59˚C and 32 cycles; Osteoprotegerin (OPG), 60˚C and 30 cycles; Receptor activator of nuclear factor kappa-B ligand (RANKL), 59˚C and 36 cycles; and Glyceraldehyde 3-phosphate dehydrogenase (GAPDH), 60˚C and 30 cycles. GAPDH was used as the internal control. PCR products were electrophoresed on1.2% agarose gel (Takara Bio Inc.) buffered with 0.5X Tris-borate-EDTA and stained with ethidium bromide (GeNet Bio Co.) after amplification. The staining bands were visualized by Gel-Doc (Bio-Rad Lab., Hercules, CA, USA). The intensities of the bands were measured and quantified using Science Lab Image Gauge (Fuji Film Co., Tokyo, Japan).

**Table 1.** Nucleotide sequences of primer used for RT-PCR.

Gene	Sequence
Alkaline phosphatase (ALP)	Forward 5′-AAG ACG TGG CGG TCT TTG C-3
	Reverse 5′-GGG AAT CTG TGC AGT CTG TG-3′
Bone sialoprotein (BSP)	Forward 5′-ACC GGC CAC GCT ACT TTC TTT AT-3′ Reverse 5′-TCC TCG TCG CTT TCC TTC ACT TT-3′
Dentin sialophosphoprotein (DSPP)	Forward 5′-CGA CCC TTG TCC AGG A-3′ Reverse 5′-CAT GGA CTC GTC ATC GAA-3′
Dentin matrix acidic phosphoprotein 1 (DMP-1)	Forward 5′-CGA GTC TCA GGA GGA CA-3′ Reverse 5′-CTG TCC TCC TCA CTG GA-3′
Osteocalcin (OCN)	Forward 5′-TGA GGA CCC TCT CTC TGC TC-3′ Reverse 5′-GAG CTC ACA CAC CTC CCT GT-3′
Osteonectin (ON)	Forward 5′-ATT TGA GGA CGG TGC AGA GG-3′ Reverse 5′-TCT CGT CCA GCT CAC ACA CCT-3′
Collagen type I (Col I)	Forward 5′-ATT CGG AGC TCA AGA TGT AA-3′ Reverse 5′-CAG TCA AGT CCT AGC CAA AC-3′
Runt-related transcription factor 2 (Runx-2)	Forward 5′-GCAGTGCCCCGATTGAGG-3′ Reverse 5′-CATACTGGGATGAGGAATGC-3′
Osteoprotegerin (OPG)	Forward 5′-CAGAGACTAATAGATCAAAGGCAGG-3′ Reverse 5′-ATGAAGTCTCACCTGAGAAGAACC-3′
Receptor activator of nuclear factor kappa-Β ligand (RANKL)	Forward 5′-TATGATGGAAGGCTCATGGT-3′ Reverse 5′-TGTCCTGAACTTTGAAAGCC-3′
Glyceraldehyde 3-phosphate dehydrogenase (GAPDH)	Forward 5′-CCA TGG AGA AGG CTG GG-3′ Reverse 5′-CAA AGT TGT CAT GGA TGA CC-3′

### ALP staining assays and Alizarin red S staining

To identify the formation of mineralized nodules, the MC3T3-E1 cells plated on the Ti discs (1.5 × 10^5^ cells/ml) were incubated for 4, 7 and 10 days in differentiation medium with or without RA. For ALP staining, the cells were fixed in 4% paraformaldehyde, rinsed 3 times with 1X TBST, and then treated with an ALP substrate solution (NBT/BCIP Solution; Roche Applied Science, Indianapolis, IN, USA) for 1 h at 37°C. For Alizarin Red S staining, the cells were fixed in 4% paraformaldehyde (Sigma-Aldrich Chemical Co.) and stained with 2% Alizarin Red S (pH 4.2) (Sigma-Aldrich Chemical Co.) for 15 min.

Mineralized nodules were observed using a stereoscopic micro­scope (Stemi 2000-C, Carl Zeiss, Oberkochen, Germany). For quantitative analysis, the stains were extracted with 10% cetylpyridium chloride (Samchun chemical Co., Seoul, Republic of Korea) in 10 mM sodium phosphate (pH 7.0) for 15 min. Alizarin red stain and ALP stain were then quantified by measuring absorbance at a wavelength of 562 nm and 540 nm, respectively, using a microplate reader (BioTek Instruments Inc., Winooski, VT, USA).

### Protein extraction and western blot analysis

Total protein was extracted from MC3T3-E1 cells on the Ti discs using a NP-40 lysis buffer. After electrophoresis and transferring protein, the membrane was blotted with the primary antibodies for 16 h at 4°C, such as 1:2000 of anti-rabbit Runx-2 (SantaCruz Biotechnology Inc., Dallas, TX, USA), 1:2000 of anti-goat OPG (SantaCruz Biotechnology Inc.), 1:1000 of anti-mouse RANKL (Novus Biological Inc., Centennial, CO, USA) and 1:2,500 of anti-mouse β-actin (Santa Cruz Biotechnology Inc.). After washing, the membrane was blotted with 1:5000 of either horseradish peroxidase (HRP)-conjugated goat anti-rabbit or mouse-IgG (Enzo Life Sciences Inc., New York, NY, USA) and HRP- conjugated donkey anti-goat-IgG (SantaCruz Biotechnology Inc.). The developing was performed using X-ray film (Fuji Film Co.) after detection using an ECL solution (Merck Millipore, Burlington, MA, USA). The density of the expressed bands was measured using Science Lab Image Gauge (Fuji Film Co.).

### Statistical analysis

All experiments were carried out in triplicate. All data is reported the mean and standard deviation determined using SPSS 12.0 (SPSS Inc., Chicago, IL, USA). The significant differences (*p* < 0.05) were determined using an independent samples t-test.

## Results

### Effect of RA on MC3T3-E1 cells viability on Ti surface during differentiation

**Figure 1.**
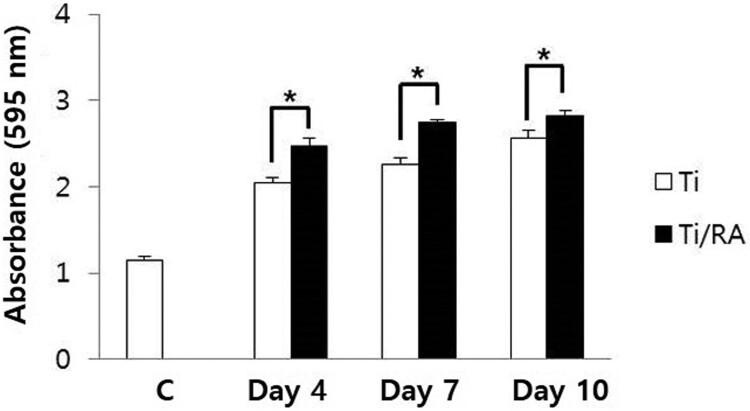
Effect of RA on viability of MC3T3-E1 cells on Ti surface during differentiation. The viability of MC3T3-E1 cells on Ti discs treated with or without 14 µg/ml RA was confirmed by an MTT assay. During the differentiation of MC3T3-E1 osteoblastic cells into osteoblasts on the Ti surface, the survival rate of RA-treated cells was increased compared to RA untreated cells (*p* < 0.05).

During the differentiation of MC3T3-E1 osteoblastic cells into osteoblasts on the Ti surface, the survival rate of RA-treated cells was increased compared to RA untreated cells (*p* < 0.05, Figure 1).

### Effect of RA on expression of non-collagenous and collagenous mRNA in MC3T3-E1 cells on Ti surface during differentiation

**Figure 2.**
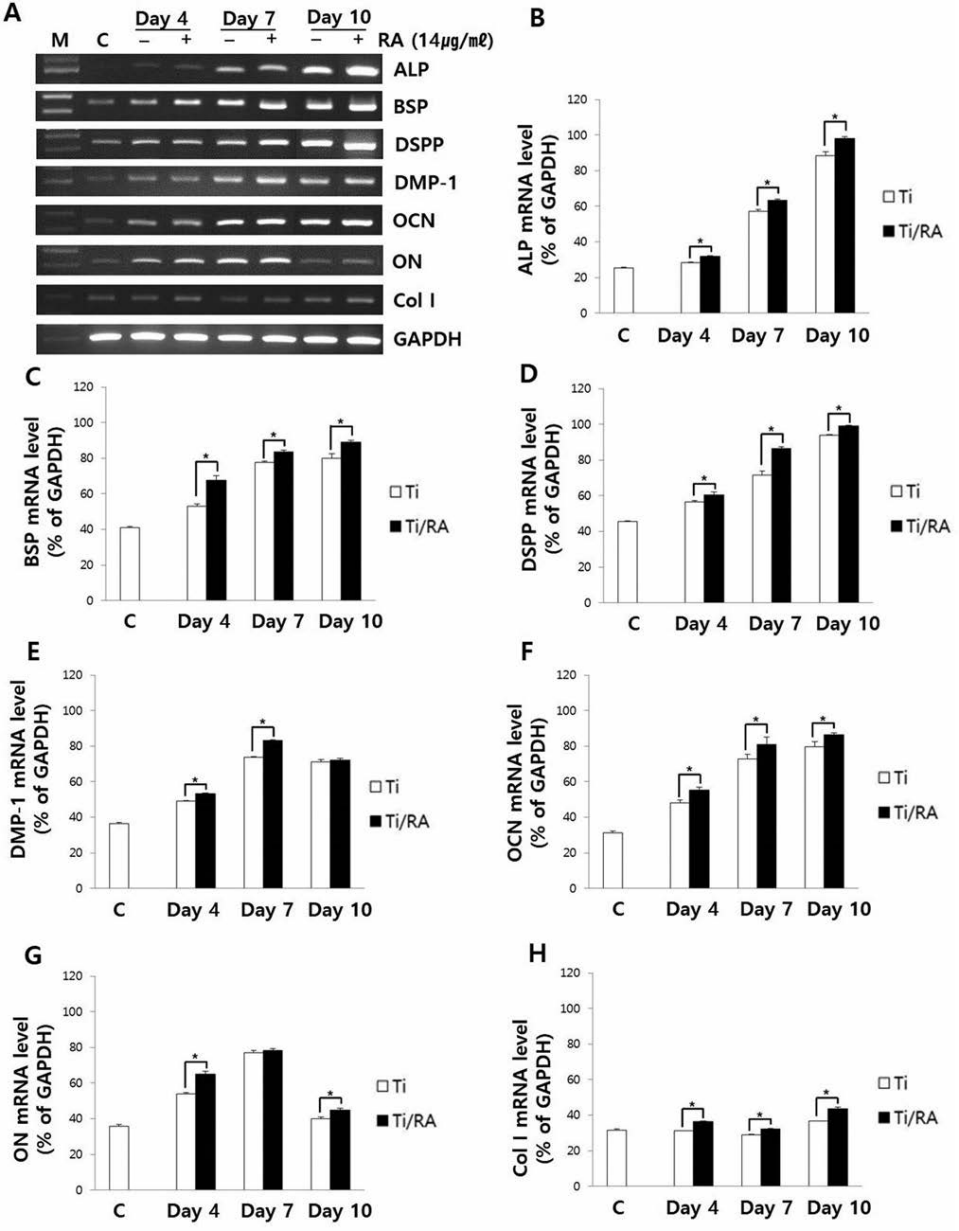
Effect of RA on expression of non-collagenous and collagenous mRNA in MC3T3-E1 cells on Ti surface during differentiation. (A) RT-PCR analysis showing the levels of non-collagenous and collagenous mRNA expression. Results of quantitative analysis of ALP (B), BSP (C), DSPP (D), DMP-1 (E), OCN (F), ONG (G) and Col I (H) expression in MC3T3-E1 cells with or without RA treatment. Non-collagenous and collagenous mRNA expressions of RA-treated cells were higher than those of untreated cells during the differentiation of MC3T3-E1 osteoblastic cells into osteoblasts (*p* < 0.05).

RT–PCR analysis showed that mRNA expression of ALP, BSP, DSPP, OCN and Col I increased with time in all RA-treated and untreated cells, except DMP-1 and ON, as well as mRNA expression of RA-treated cells was higher than that of untreated cells during the differentiation of MC3T3-E1 osteoblastic cells into osteoblasts on the Ti surface (*p* < 0.05, Figure 2).

### Effect of RA on mineralization of MC3T3-E1 cells on Ti surface during differentiation

**Figure 3.**
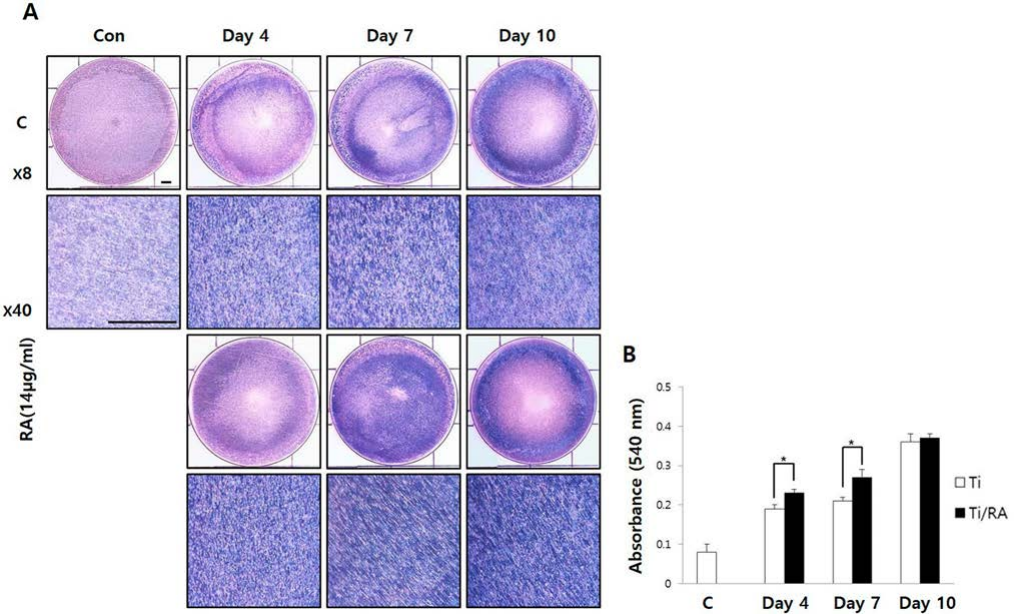
Effect of RA on mineralization through ALP staining in MC3T3-E1 cells on Ti surface during differentiation. (A) ALP staining of MC3T3-E1 cells on Ti discs. (B) Results of quantitative analysis of mineralization in MC3T3-E1 cells on Ti discs with or without RA treatment. During the differentiation of MC3T3-E1 osteoblastic cells into osteoblasts on the Ti surface, the activities of ALP of RA-treated cells were increased compared to untreated cells (*p* < 0.05).

**Figure 4.**
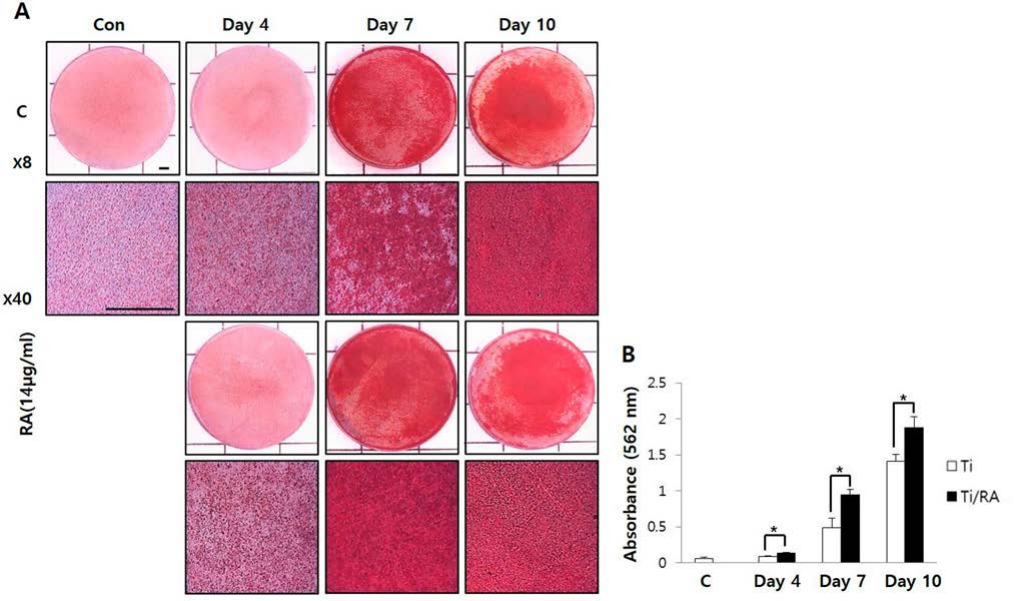
Effect of RA on mineralization through Alizarin Red S staining in MC3T3-E1 cells on Ti surface during differentiation. (A) Alizarin Red S staining of MC3T3-E1 cells on Ti discs. (B) Results of quantitative analysis of mineralization in MC3T3-E1 cells on Ti discs with or without RA treatment. The formation of calcified nodules in RA-treated cells on Ti surface was increased compared to those of untreated cells during differentiation (*p* < 0.05).

As MC3T3-E1 cells differentiated on the Ti surface, the increase in ALP activity and mineralization was observed as time increased in both RA-treated and untreated cells, as well as increased ALP activity and mineralization of RA-treated MC3T3-E1 cells were confirmed in comparison with untreated cells (*p* < 0.05, Figures 3 and 4). As a result of ALP staining, the activity of ALP of RA-treated MC3T3-E1 cells on Ti surface was increased (*p* < 0.05, Figure 3(B)). As shown in Figure 4(B), Alizarin Red S staining, specially combined with calcium, and the formation of calcified nodules in RA-treated cells on Ti surface increased compared to untreated cells (*p* < 0.05).

### Effect of RA on Runx-2, OPG and RANKL expression in MC3T3-E1 cells on Ti surface during differentiation

**Figure 5.**
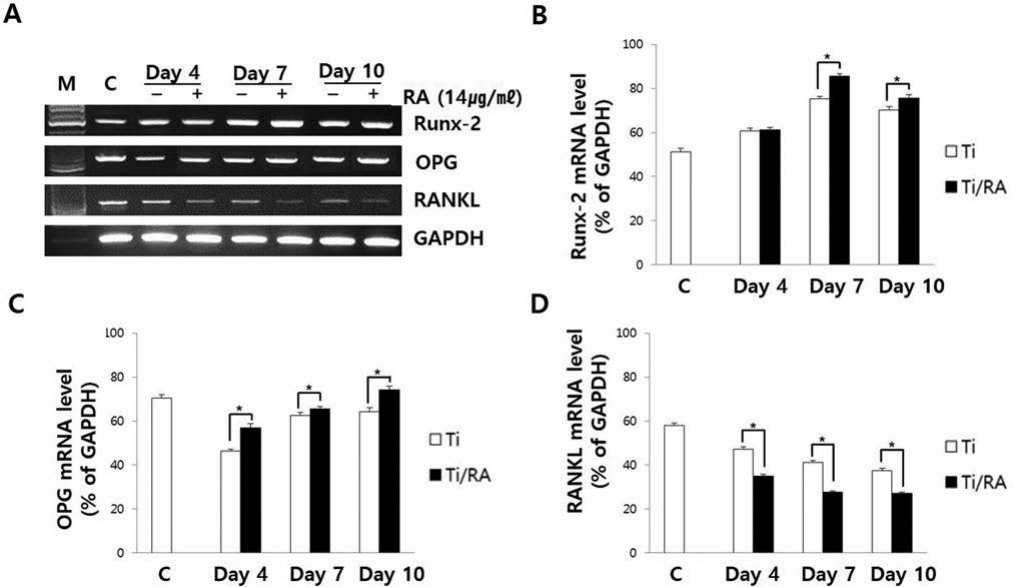
Effect of RA on mRNA expression of Runx-2, OPG and RANKL in MC3T3-E1 cells on Ti surface during differentiation. (A) RT-PCR analysis showing the levels of Runx-2, OPG and RANKL mRNA expression. Results of quantitative analysis of Runx-2 (B), OPG (C) and RANKL (D) expression in MC3T3-E1 cells with or without RA treatment. During the differentiation of MC3T3-E1 osteoblastic cells to osteoblasts on Ti surface, RA increased the mRNA expression of Runx-2 and OPG in MC3T3-E1 cells, but decreased RANKL mRNA expression (*p* < 0.05).

**Figure 6.**
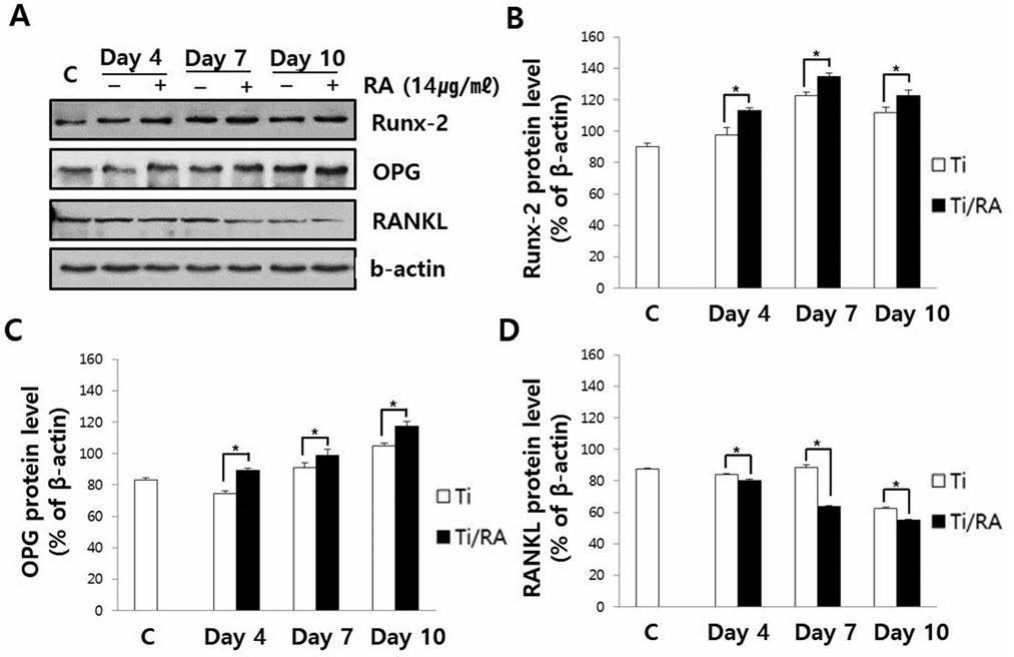
Effect of RA on protein expression of Runx-2, OPG and RANKL in MC3T3-E1 cells on Ti surface during differentiation. (A) Runx-2, OPG and RANKL protein levels were assayed by Western blotting. (B) Results of quantitative analysis of Runx-2 (B), OPG (C) and RANKL (D) protein levels in MC3T3-E1 cells with or without RA treatment. RA increased the Runx-2 and OPG protein expression in MC3T3-E1 cells on Ti surface, but decreased RANKL protein expression (*p* < 0.05).

**Figure 7.**
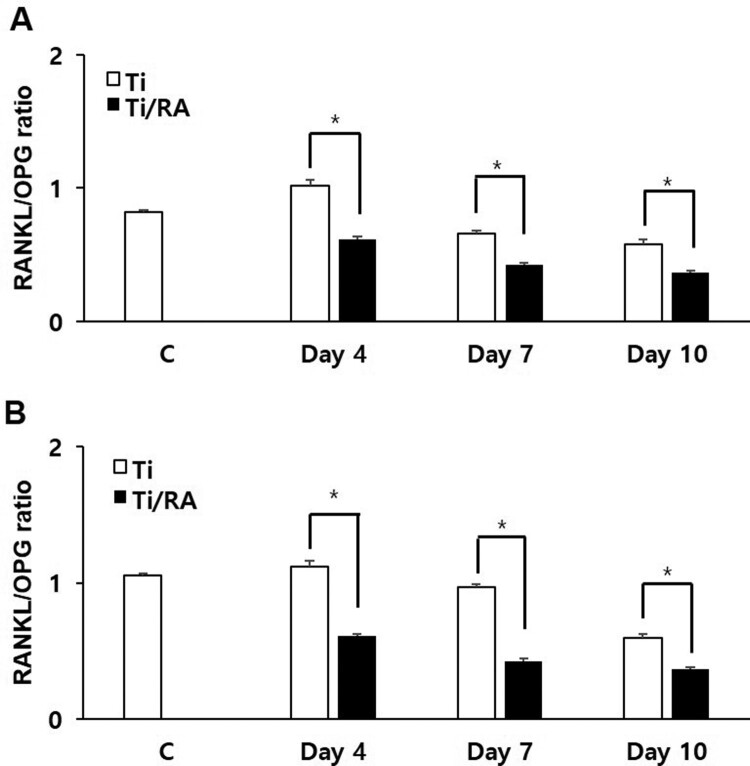
Effect of RA on the ratio of RANKL to OPG in MC3T3-E1 cells on Ti surface during differentiation. RA decreased the ratio of RANKL to OPG mRNA (A) and protein (B) during the differentiation of MC3T3-E1 cells on Ti surface (*p* < 0.05).

During the differentiation of MC3T3-E1 cells on Ti surface, the mRNA and protein expression of Runx-2 and OPG in RA-treated cells were increased, but those of RANKL was decreased (*p *< 0.05, Figures 5 and 6). The ratio of RANKL to OPG in mRNA and protein expression of RA-treated MC3T3-E1 cells was significantly lower than that of untreated cells (*p* < 0.05, Figure 7).

## Discussion

**Figure 8.**
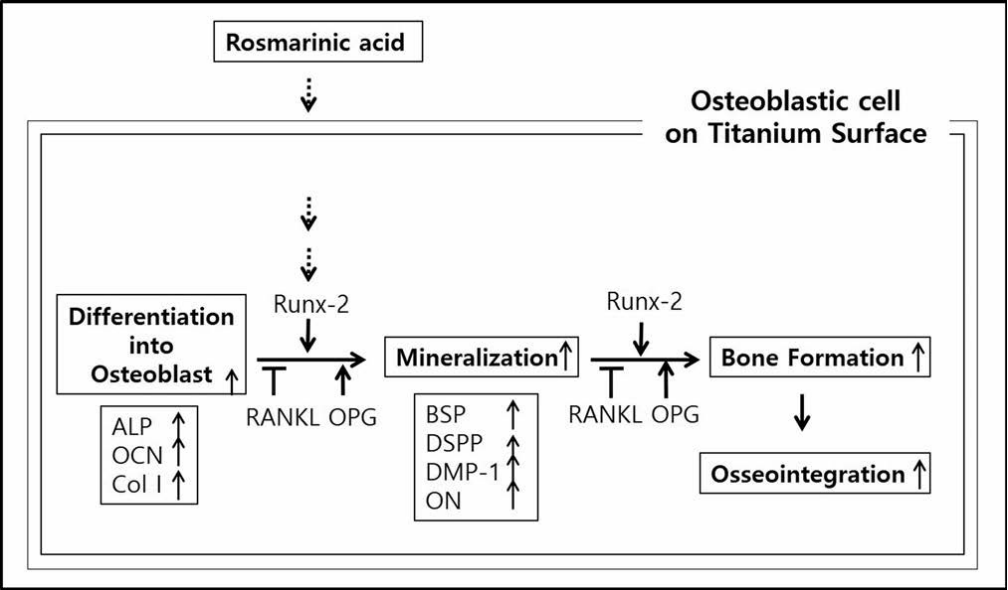
Simple schematic diagram of RA effects in osseointegration of MC3T3-E1 cells on Ti surface. RA enhances osseointegration by increasing differentiation, mineralization, and bone formation through stimulation of Runx-2 and OPG expression and inhibition of RANKL expression in the RANKL/RANK/OPG pathway during the differentiation of MC3T3-E1 osteoblastic cells to osteoblasts on the Ti surface.

RA is a plant-derived phytochemical with low toxicity and side effects and is known to have various effects that can be applied as a therapeutic substance for acute and chronic diseases (Kim et al. 2008; Moon et al. 2010; Lee and Han 2012; Kang et al. 2017; Kim et al. 2018). We investigated the RA effects of cell viability, differentiation into osteoblasts and mineralization of MC3T3-E1 osteoblastic cells on the Ti surface, during differentiation and confirmed the applicability of RA as a functional and therapeutic substance to improve osseointegration between Ti and osteoblasts. The mRNA expression of ALP, BSP, DSPP, DMP-1, OCN, ON and Col I are biomarkers that are associated with osteoblast differentiation and mineralization into of bone formation (Choi et al. 2016; Jeong and Jeong 2016). During differentiation of osteoblastic cells into osteoblasts, expression of genes for the formation of bone matrix such as ALP, Col I and OCN is increased (Choi et al. 2016). In particular, ALP is an essential enzyme expressed early-stage in differentiation and its expression is increased during differentiation, which is used as a marker for an early-stage differentiation of osteoblast (Lee et al. 2015). Col I, along with ALP, is an important protein that increases expression early-stage in differentiation and induces differentiation but decreases Col I mRNA expression during mineralization in MC3T3-E1 cells (Choi et al. 2016). OCN is expressed in the late-stage of differentiation into osteoblasts, used as a marker for a late-stage differentiation and related to bone matrix formation (Lee et al. 2015; Choi et al. 2016). RA significantly increased the expression of ALP, Col I and OCN (Figure 2), indicating that RA effectively induces both early and late differentiation of MC3T3-E1 osteoblastic cells into osteoblasts (Figure 5). The secreted organic matrix is mineralized by calcium deposition in the late-stage of osteoblast differentiation (Choi et al. 2016). BSP, DSPP, DMP-1 and ON are associated with mineralization of bone matrix (Oldberg et al. 1988; Hunter and Goldberg 1994; Choi et al. 2018). BSP is an important protein in mineralized tissue that binds hydroapatite and acts as a nucleator for the formation of apatitic crystals early-stage in mineralization, and also regulates the direction of ribbon-like apatite crystal growth during mineralization (Oldberg et al. 1988; Hunter and Goldberg 1994; Choi et al. 2018). DSPP is expressed in early-stage of hydroxyapatite formation and dentin mineralization in osteogenic cells such as MC3T3-E1 osteoblastic cells and odontoblast (Saito et al. 1997; Choi et al. 2016). DMP-1 increases expression during bone nodules formation and mineralization, and serves as a nucleator for the formation of hydroxyapatite (He et al. 2003). ON modulates bone matrix assembly and is responsible for the forms, remodeling and maintains of the early bone matrix (Lian et al. 1999; Delany et al. 2000; Anne et al. 2003). The secreted extracellular organic matrix is mineralized by calcium deposition in the late-stage of osteoblast differentiation, and increased mineral deposition is an important factor indicating the viability of osteoblasts on biomaterials for implants (Choi et al. 2016; Jeong and Jeong 2016). Treatment of RA significantly increased BSP, DSPP, DMP-1 and ON expression related to mineralization of bone matrix (Figure 2) and mineralization by calcium deposition of organic matrix secreted by osteoblasts (Figures 3 and 4). The RA-treated MC3T3-E1 osteoblastic cells on Ti surface significantly increased the proliferation, mitosis and differentiation through the cascade of the FAK/Grab2/Ras/ERK1/2 signaling pathway (data not shown), and as a result, Runx-2 mRNA and protein expression were significantly increased (Figures 5 and 6). Runx-2 is an important transcription factor related to differentiation of pre-osteoblastic cells into osteoblast and is activated through ERK1/2 signaling (Choi et al. 2018). OPG is a decoy receptor for RANKL produced in osteoblast lineage cells and is an antagonist of RANKL (Xu and Jiang 2010; Wachi et al. 2015). OPG inhibits the differentiation and development of osteoclast by interfering with the binding of RANKL and its receptor, RANK, and a lack of OPG induces severe osteopenia and osteoclastogenesis (Xu and Jiang 2010). RANKL is an important factor for osteoclastogenesis produced by osteoblast, and its production is increased by LPS, 1a25-dihydroxyvitamin D3 (Vitamin D3) and Ti ions, which are factors that stimulate bone resorption (Wachi et al. 2015; Lee et al. 2016). Expression of OPG and RANKL through the RANKL/RANK/OPG pathway is an important regulator for bone metabolism (Xu and Jiang 2010) and is essential for the differentiation and development of osteoclasts that contribute to pathological bone resorption (Wachi et al. 2015). The ratio of RANKL to OPG is an important factor in determining bone density in normal and diseased states, and bone resorption progresses when it increases (Wachi et al. 2015). In the RA-treated MC3T3 cells on Ti surface, Runx2 and OPG increased and RANKL decreased (Figures 5 and 6), and it was confirmed that the ratio of RANKL to OPG significantly decreased during the differentiation (Figure 7). These results are consistent with studies showing the inhibitory effect of RA on metastasis from breast cancer cells to bone tissue (Xu and Jiang 2010) and RANKL-induced osteoclast differentiation and function (Lee et al. 2015) Therefore, RA enhances cell viability, differentiation, mineralization through stimulation of Runx-2 and OPG mRNA and protein expression and inhibition of RANKL expression during the differentiation of MC3T3-E1 osteoblastic cells into osteoblasts on the Ti surface (Figure 8).

## Conclusion

RA should be applied as an effective functional and therapeutic substance to enhance osseointegration of osteoblast cells by increasing cell viability, differentiation, mineralization, and bone formation through stimulation of Runx-2 and OPG expression and inhibition of RANKL expression in the RANKL/RANK/OPG pathway during the differentiation of MC3T3-E1 osteoblastic cells into osteoblasts on the Ti surface.
